# *PEG3* mutation is associated with elevated tumor mutation burden and poor prognosis in breast cancer

**DOI:** 10.1042/BSR20201648

**Published:** 2020-08-11

**Authors:** Min Zhang, Jin Zhang

**Affiliations:** 1 The Third Department of Breast Cancer, Tianjin Medical University Cancer Institute and Hospital, National Clinical Research Center for Cancer, Tianjin 300060, China; 2 Key Laboratory of Cancer Prevention and Therapy, Tianjin 300060, China; 3 Tianjin’s Clinical Research Center for Cancer, Tianjin 300060, China; 4 Key Laboratory of Breast Cancer Prevention and Therapy, Tianjin Medical University, Ministry of Education, Tianjin 300060, China

**Keywords:** breast cancer, high tumor mutation burden, immunotherapy, inferior prognosis, PEG3 mutation

## Abstract

**Background:** Breast cancer is the second most common malignancy in women and considered as a severe health burden. *PEG3* mutations have been observed in several cancers. However, the associations of *PEG3* mutation with tumor mutation burden (TMB) and prognosis in breast cancer have not been investigated. **Methods:** In our study, the somatic mutation data of 986 breast cancer patients from The Cancer Genome Atlas (TCGA) were analyzed. **Results:** It showed that *PEG3* had a relatively high mutation rate (2%). After calculated the TMB in *PEG3* mutant and *PEG3* wild-type groups, we found the TMB value was significantly higher in *PEG3* mutant samples than that in *PEG3* wild-type samples (*P* = 5.6e-07), which was independent of the confounding factors including age, stage, mutations of *BRCA1, BRCA2* and *POLE* (odd ratio, 0.45; 95% CI, 0.20–0.98; *P*=0.044). Survival analysis revealed that *PEG3* mutant samples had inferior survival outcome compared with the *PEG3* wild-type samples after adjusted for the confounding factors above (hazard ratio, 0.27; 95% CI: 0.12–0.57; *P*<0.001). **Conclusion:** These results illustrated that *PEG3* mutation was associated with high TMB and inferior prognosis, suggesting *PEG3* mutation might play a guiding role in prognosis prediction and immunotherapy selection in breast cancer.

## Introduction

Breast cancer is recognized as the second most common malignancy with the feature of distinct metastasis, which involves in liver, lung, lymph nodes and bone marrow [1,2]. Almost one in eight women is diagnosed with breast cancer in America, and the incidence rate increases with age [3,4]. Breast cancer results in 14% of death among women worldwide, which is considered as the second major cause for cancer death in women [5]. The treatment options consist of surgery, molecular treatment, chemotherapy, radiation therapy and immunotherapy [6,7]. However, the prognosis of breast cancer patients is not optimistic. Although patients have received corresponding treatment, the recurrence rate is still increased steadily, which may be attributed to the tumor diameter and nodal status [8]. Thus, more investigations on the underlying molecular mechanism may be significant for timely surveillance as well as improved prognosis of breast cancer.

Multiple factors have been identified to be associated with the occurrence of breast cancer, such as age, history of cancer, menarche, childbearing, history of mammary gland diseases and race [9]. Besides, genetic factors are also proved to contribute to the progression of breast cancer [10]. Xu et al. found nerve guidance factor 4 (*NTN4*), which played a crucial role in regulating the migration and invasion of breast cancer cells, presented decreased expression level in breast cancer [11]. *Dyrk1B*, encoding the serine/threonine kinase implicated in modulation of cell proliferation and cancer progression, was suggested to be involved in breast cancer progression and led to poor prognosis [12]. Long et al. found that low-penetrance variants in genes *PALB2, CHEK2, BRCA1*, and *BRCA2* might be related to the risk of breast cancer [13]. *PEG3*, located on 19q13.4, is an imprinted gene and encodes C2H2 zinc-finger protein [14,15]. The dysfunctions of *PEG3* frequently occur in several cancers. Compared with the normal tissues, decreased expressions of *PEG3* were identified in 18 cancer types [16]. The abnormity of *PEG3* methylation was associated with elevated risk of invasive cervical cancer [17]. Loss of *PEG3* was found to result in the pathogenesis of ovarian cancer [18]. However, as far as we know, the mutation of *PEG3* was seldom studied in breast cancer.

Immunotherapy provides new option and direction for cancer treatment. But whether immunotherapy is the optimal choice for individual patient depends largely on their response to anti-PD-1/PD-L1 treatment. The ratio of PD-L1 positive tumor cells, a common biomarker for immunotherapy response, still faces many challenges in accuracy [19]. Tumor mutation burden (TMB), related to the neoantigen number in tumors, plays a crucial role in the effects prediction of immune checkpoint inhibitors and is an ideal biomarker for immunotherapy response [20,21]. TMB has been proved to be related to gene mutations in several cancers. For example, Chen et al. found MUC16 was associated with elevated TMB in gastric cancer [22]. Mutations of DNA repair genes were found to be associated with increased TMB in ovarian carcinoma [23]. But to our knowledge, no researches have reported the relationship of PEG3 mutation with TMB in breast cancer yet.

In the present study, we analyzed the somatic mutation data of breast cancer and explored the association between PEG3 mutation and TMB. Then, the survival and COX regression analyses were performed to further investigate the relationship between PEG3 mutation and prognosis of breast cancer patients. Given these findings, our study aimed to elaborate the guiding role of PEG3 mutation in prognosis prediction and immunotherapy selection in breast cancer.

## Materials and methods

### Data source

The somatic mutation data of 986 patients were obtained from The Cancer Genome Atlas (TCGA, www.cancergenome.nih.gov), including 1268 samples. Among them, 975 patients had complete survival information.

### Extraction of mutation signature

The SignatureAnalyzer [24] was used for extraction of mutation signature from somatic mutation data of maf files, which was based on Bayesian-based nonnegative matrix factorization method.

### Calculation of TMB

TMB was defined as the average number of mutations per megabase in the genome. Based on the maf file, mutations of patients were calculated and TMB was expressed as the ratio of mutation number to exon length.

### Statistical analysis

#### Distribution of TMB in *PEG3* mutant and *PEG3* wild-type groups

The breast cancer samples were divided into PEG3 mutant and PEG3 wild-type groups. The difference of TMB between them was analyzed by two-sided *t* test, with *P*<0.05 as threshold. The effects of multiple factors on TMB were analyzed by Wilcoxon rank-sum test, and *P*<0.05 was considered statistically significant.

#### Survival analysis

We used survminer package in R software to draw the survival curve. Survival package in R software and COX regression analysis were adopted to analyze the effects of confounding factors on survival, such as age and tumor stage. As the mutations of BRCA1, BRCA2 and POLE are involved in the damage and repair of genome, which contribute to mutations of other genes [22], in the present study BRCA1, BRCA2 and POLE mutations were also included into the factors. Gender was excluded because breast cancer was mainly occurred in women.

### Analysis of significantly mutated genes (SMG)

MutSigCV algorithm was performed to analyze the significantly mutated genes (SMG) according to previous study [25]. Mutations in tumor are divided into driver mutation that confers a selective growth advantage to cells and passenger mutation that is accompanied with driver mutation [26]. Thus, researches on the driver mutation is significant for comprehensive understanding of tumor progression mechanism. Herein, MutSigCV was used to analyze the driver genes in breast cancer samples.

## Results

### The mutation spectrum in breast cancer

**Figure 1:**
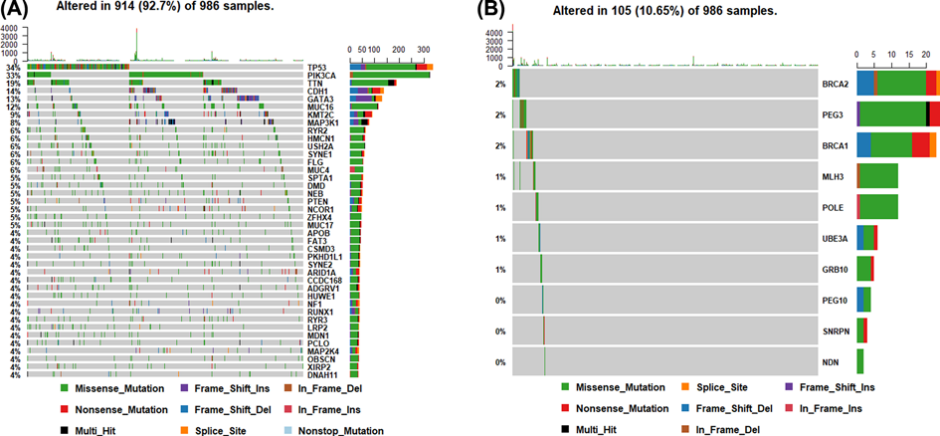
Gene mutation rates and classifications across each breast cancer sample (**A**) Mutation rates and classifications of the top 40 mutated genes. (**B**) Mutation rates and classifications of imprinted genes (*PEG3, UBE3A, GRB10, PEG10, SNRPN, NDN*) and genes that might affect the mutation rates of the genome (*BRCA1, BRCA2, POLE, MLH3*).

After analyzing the mutation data, we found missense mutation accounted for a large proportion in the variant classification among breast cancer samples. The main variant type was single-nucleotide polymorphism (SNP), with C > T as the major form (Supplementary Figure S1). We calculated the mutation rates of the top 40 mutated genes (Figure 1A), imprinted genes (PEG3, UBE3A, GRB10, PEG10, SNRPN, NDN) and genes that might affect the mutation rates of the genome (BRCA1, BRCA2, POLE, MLH3) (Figure 1B) respectively, and the result revealed that PEG3 and BRCA2 presented relatively high mutation rates in the breast cancer samples.

### Mutations of BRCA2, BRCA1, MLH3 and POLE is associated with high mutation frequencies in breast cancer patients

As shown in Figure 1B, the mutation frequencies exhibited high levels in samples with mutations of BRCA2, BRCA1, MLH3 and POLE, further illustrating that they might be associated with the stability of genome.

### PEG 3 mutation is associated with high TMB in breast cancer patients

**Figure 2:**
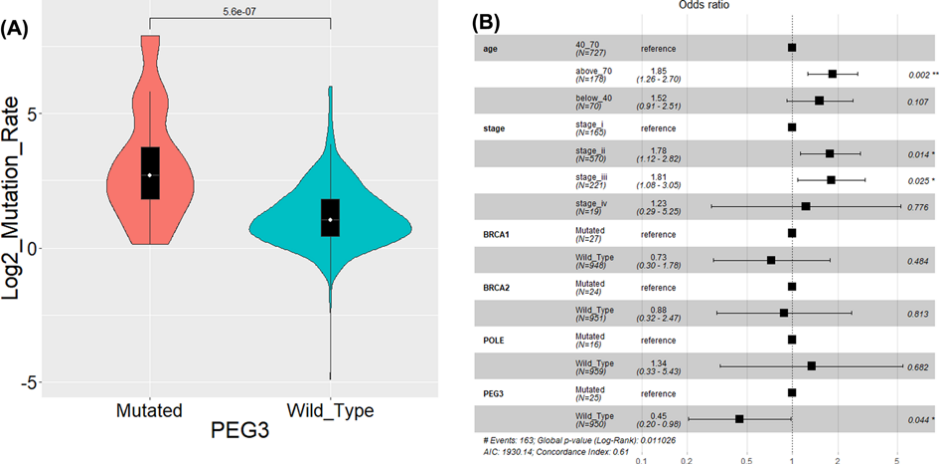
PEG 3 mutation is associated with high TMB in breast cancer (**A**) Mutation rates in *PEG3* mutant and *PEG3* wild-type groups. (**B**) Association of *PEG3* mutation with TMB after adjusted for age, stage, mutations of *BRCA1, BRCA2* and *POLE.* TMB: tumor mutation burden.

The breast cancer samples were assigned into PEG3 mutant and PEG3 wild-type groups, and TMB in the two groups were calculated. As shown in Figure 2A, TMB in PEG3 mutant group was significantly elevated compared with PEG3 wild-type group (*P*=5.6e-07). To eliminate the effects of other factors, age, stage, mutations of BRCA1, BRCA2 and POLE were considered into the multivariate regression analysis. It was found that the TMB was still significantly lower in PEG3 wild-type group than that in PEG3 mutant group (odd ratio, 0.45; 95% CI, 0.20–0.98; *P*=0.044; Figure 2B).

### PEG3 mutation is associated with inferior prognosis in breast cancer

**Figure 3:**
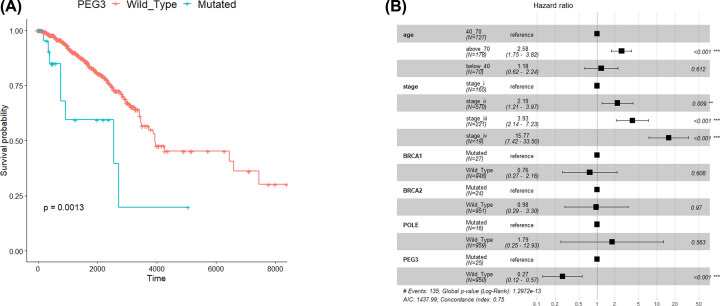
*PEG3* mutation is associated with inferior prognosis in breast cancer (**A**) Survival curves of *PEG3* mutant and *PEG3* wild-type groups. (**B**) Association of *PEG3* mutation with survival status after adjusted for age, stage, mutations of *BRCA1, BRCA2*, and *POLE.*

The survival curves of PEG3 mutant and PEG3 wild-type groups were shown in Figure 3A. It was revealed that PEG3 mutant samples had inferior overall survival compared with the PEG3 wild-type samples (*P*=0.0013). After including several confounding factors (age, stage, mutations of BRCA1, BRCA2, and POLE) into the COX regression model, the overall survival in PEG3 wild-type samples remained better than that in PEG3 mutant samples (hazard ratio, 0.27; 95% CI: 0.12–0.57; *P*<0.001, Figure 3B).

### Signatures 2, 1, and 10 account for high percentages in breast cancer samples

**Figure 4:**
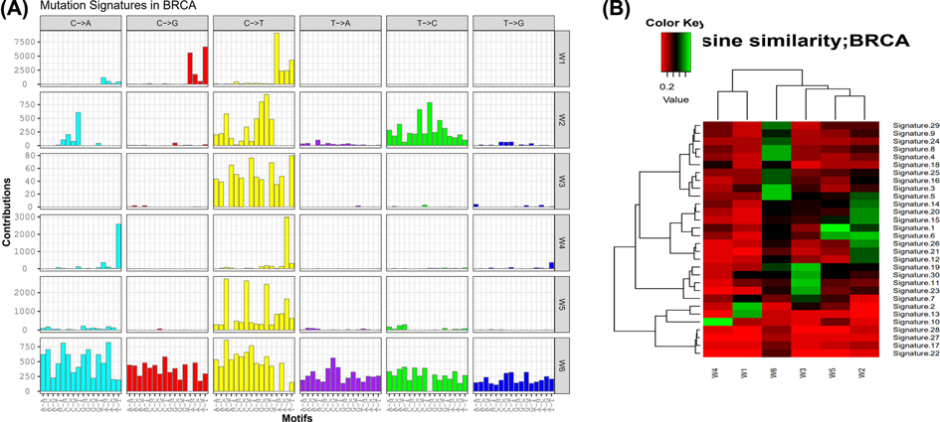
High similarities of W1-W6 extracted from breast cancer samples with Signature2, Signature6, Signature19, Signature10, Signature1, Signature5 from COSMIC database are identified (**A**) The six mutation signatures (W1-W6) extracted from breast cancer samples. (**B**) Similarities between the signatures we extracted and those included in COSMIC database.

After analyzed by SignatureAnalyzer, 6 mutation signatures were extracted and named as W1, W2, W3, W4, W5 and W6, respectively (Figure 4A). Then, the 6 mutation signatures were compared with those included in COSMIC database (https://cancer.sanger.ac.uk/cosmic/). As shown in Figure 4B, there were high similarities observed between W1 and Signature2, W2 and Signature6, W3 and Signature19, W4 and Signature10, W5 and Signature1, W6 and Signature5 respectively.

**Figure 5:**
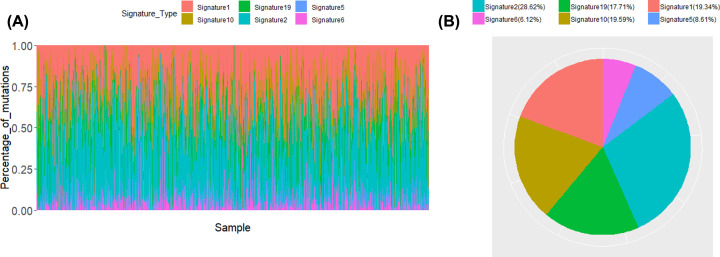
Signatures 2, 1 and 10 account for high percentages in breast cancer samples (**A**) The proportion of each signature in individual breast cancer sample. (**B**) The proportion of each signature in overall breast cancer samples.

The proportion of each signature in breast cancer samples was further calculated. As shown in Figure 5, signatures 1, 2 and 10 accounted for large percentages in both individual and whole breast cancer samples. These signatures were mainly associated with biological functions of mRNA-editing enzyme overactivity, accumulation of C > T CpG, and defects in DNA proofreading [22].

### TP53 and GATA3 may be mutation-driver genes in breast cancer

**Figure 6:**
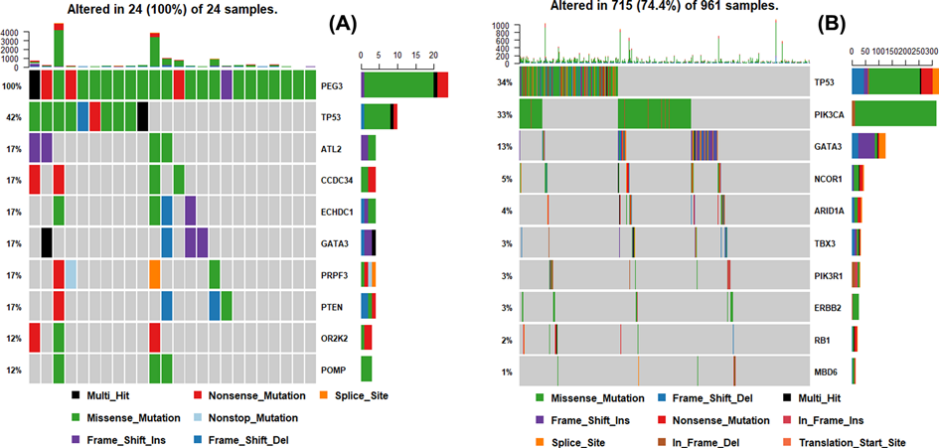
Mutation types of the top 10 SMGs across each sample in *PEG3* mutant and *PEG3* wild-type groups (**A**) Variant classification and distribution of the top 10 SMGs in *PEG3* mutant group. (**B**) Variant classification and distribution of the top 10 SMGs in *PEG3* wild-type group; SMG: significantly mutated genes.

**Figure 7:**
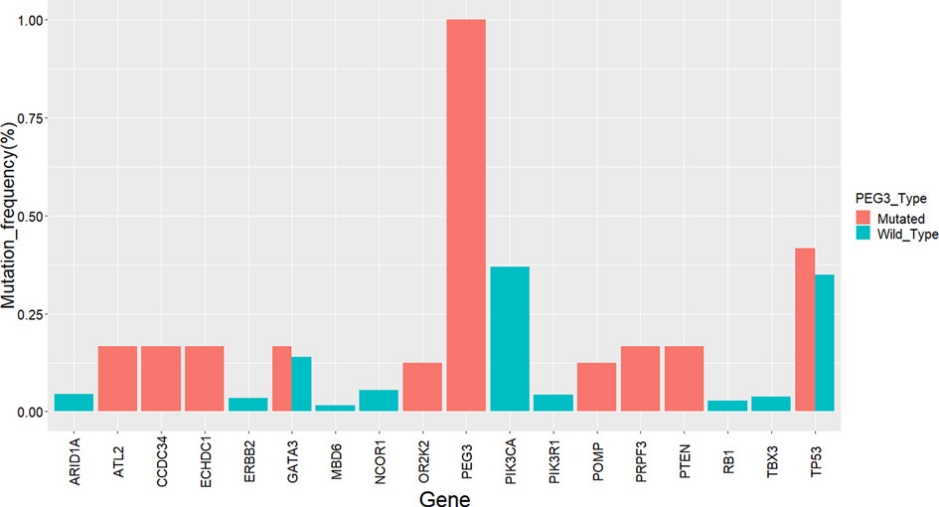
SMGs *TP53* and *GATA3* present high mutation rates in both *PEG3* mutant group and *PEG3* wild-type group SMG: significantly mutated gene.

We used the MutSigCV algorithm to analyze the SMG of PEG3 mutant and PEG3 wild-type groups. The top 10 of the SMGs were selected to further analyze their mutations in the two groups, which were shown in Figure 6A,B, respectively. The results revealed that high mutation rates of TP53 and GATA3 were observed in both of the two groups, especially in the PEG3 mutant group, suggesting they might act as mutation-driver genes in breast cancer (Figure 7).

## Discussion

Breast cancer is considered as a public health problem worldwide [27]. In the present study, we analyzed the mutation data of 986 breast cancer patients from TCGA and found that PEG3 exhibited a relatively high mutation rate among the patients. Furthermore, PEG3 mutation was significantly associated with high TMB and inferior prognosis, and the associations were independent of multiple confounding factors including age, tumor stage and mutations of BRCA1, BRCA2 and POLE.

The mutation rate of PEG3 was 2% in the breast cancer samples, which was relatively high. *PEG3*, a paternally expressed imprinted gene, encodes C2H2 zinc-finger protein and is mainly expressed multiple tissues such as brain, testis, ovary, placenta and could regulate the physiological processes related to energy homeostasis [28–30]. Perinatal growth retardation occurred in mice with PEG mutation [31]. Moreover, PEG mutations were found in several cancers. Among 98 Chinese patients with colorectal cancer, PEG3 showed a mutation frequency of 10.6% [32]. Mutation in PEG3 was identified in Opisthorchis viverrini-related cholangiocarcinoma [33]. However, the dysfunction of PEG3, especially its mutation, was rarely studied in breast cancer. Our result provided a foundation for the researches of PEG3 mutation in breast cancer.

The survival analysis revealed PEG3 mutant patients had worse survival outcome after controlled for multiple factors including age, tumor stage and mutations of BRCA1, BRCA2 and POLE. PEG3 can inhibit Wnt signaling pathway by interacting with β-catenin and induce p53-mediated apoptosis by cooperating with Siah1, and play a pivotal role in tumor suppression [34,35]. It was shown that the down-regulated expression of PEG3 protein resulted in enhanced proliferation and decreased apoptosis in glioma stem cells [34]. Wnt signaling pathway was activated in breast cancer, and the related genes of this pathway were also overexpressed [36]. It was suggested that Wnt signaling pathway involved in breast cancer through promoting tumor cell motility and metastasis [37,38]. The apoptosis mediated by p53 was found to be decreased in breast cancer [39]. Thus, it was inferred that PEG3 mutation resulted in the dysfunction of tumor suppressor PEG3, which decreased the inhibition of Wnt signaling pathway and induction of p53-mediated apoptosis, leading to the enhancement of tumor cell proliferation and metastasis. The biological processes above contributed to a poor prognosis in breast cancer patients consequently.

Through mutational signature analysis, we identified three dominant signatures among all breast cancer samples from TCGA, including signature 1, 2 and 10. Although signature 10 has not been well documented in COSMIC, both signature 1 and 2 are described as mutations related to deficiency in mismatch repair due to *in vivo* dissonance enzymes, which have been reported to be closely reported to genomic instability and tumorigenesis. In PEG3 mutant group, the TMB was significantly elevated after adjusted for multiple confounding factors, indicating PEG3 might be a vital predictor of TMB. TMB is necessary for clinical management and prediction of immunotherapy efficacy in breast cancer [40]. Although microsatellite instability-high (MSI-H) and mismatch-deficiency (MMR) were also closely focused for their guiding role in immunotherapy, their aberrant ratios were relatively low in breast cancer, making TMB a proper alternative [41]. Sun et al. demonstrated the expression levels of HER-2, ER, Ki-67 and PR acted as predictive factors of TMB [42]. APOBEC mutation was highlighted for the predictive value on TMB and responses to therapy in breast cancer [43]. It was also found that TMB with mutations of BRCA1 or BRCA2 was a prognosis signature in breast cancer, which was able to predict the treatment response [44]. However, the association between PEG3 and TMB in breast cancer has not been investigated yet. Our study elaborated the predictive role of PEG3 in TMB among breast cancer patients for the first time.

In conclusion, our study revealed PEG3 mutation was associated with high TMB and poor prognosis in breast cancer patients, which might predict the survival outcome in clinical trials.

## Supplementary Material

Supplementary Figure S1

## Abbreviations

*NTN4*: nerve guidance factor 4
SMG: significantly mutated genes
SNP: single-nucleotide polymorphism
TCGA: The Cancer Genome Atlas
TMB: tumor mutation burden
