# Supplementary material for: PEG3 mutation is associated with elevated tumor mutation burden and poor prognosis in breast cancer
Source: Biosci Rep. 2020 Aug 11;40(8):BSR20201648. doi: 10.1042/BSR20201648 (PMC7419805; doi:10.1042/BSR20201648)
Supplement: Supplementary Figure S1 [file BSR-2020-1648_supp.pdf]

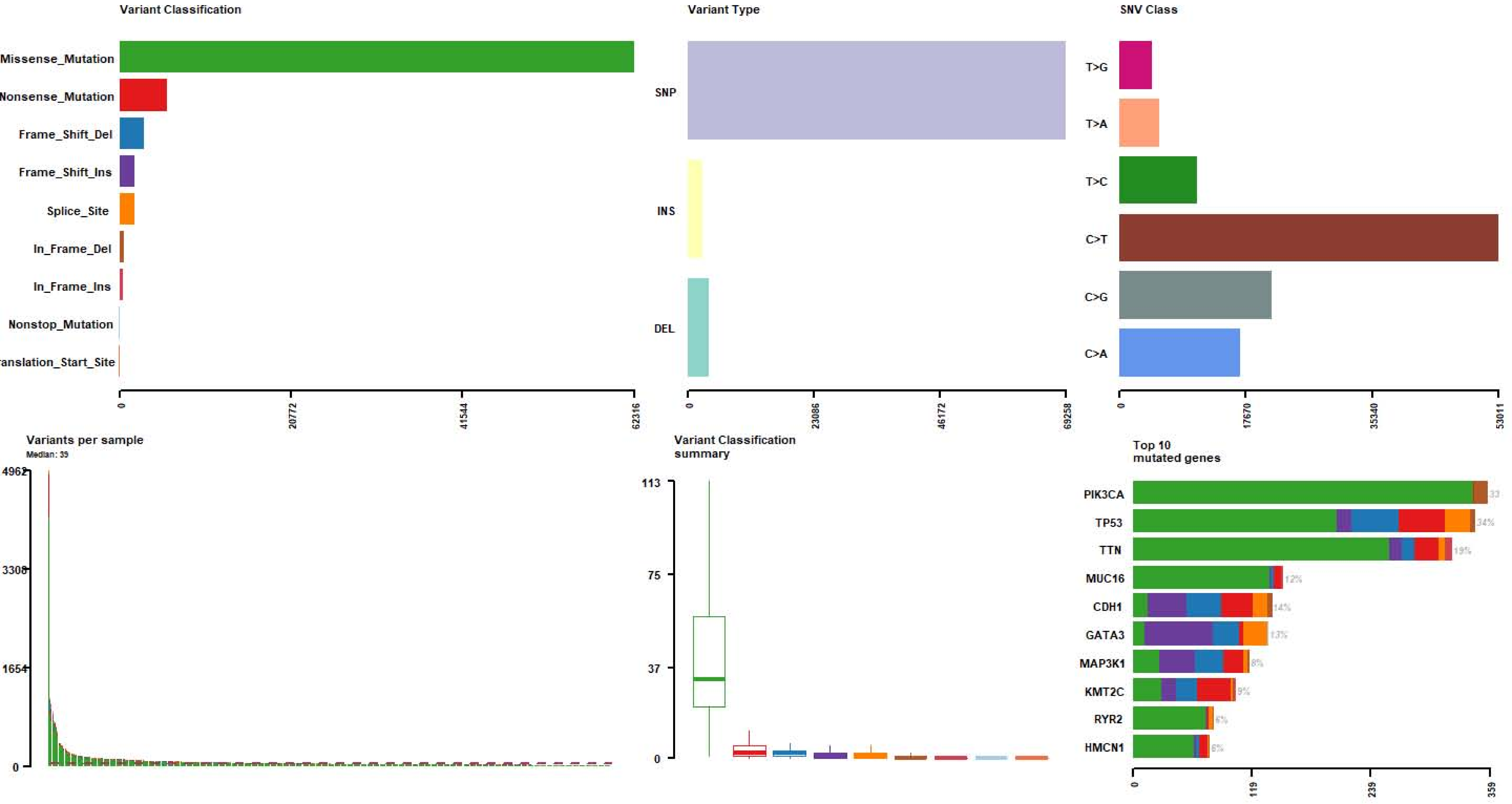
Variant Classification
Missense_Mutation
Nonsense_Mutation
Frame_Shift_Del
Frame_Shift_Ins
Splice_Site
In_Frame_Del
In_Frame_Ins
Nonstop_Mutation
ranslation_Start_Site
0
20772
41544
62316
Variant Type
SNP
INS
DEL
0
23086
46172
69258
SNV Class
T>G
T>A
T>C
C>T
C>G
C>A
0
17670
35340
53011
Variants per sample
Median: 39
4962
3308
1654
0
Variant Classification
summary
113
75
37
0
Top 10
mutated genes
PIK3CA
TP53
TTN
MUC16
CDH1
GATA3
MAP3K1
KMT2C
RYR2
HMCN1
33
34%
19%
12%
14%
13%
8%
9%
6%
6%
0
119
239
359

Figure. S1 Mutational landscape of breast cancer samples from TCGA, including variant classification (upper left), variant type (upper middle), single nucleotide variant type (upper right), variant number across all breast cancer samples (bottom left), number of breast cancer samples with specific variant classification (bottom middle), and the top 10 most frequently mutated genes (bottom right).
